# Time series cluster analysis reveals individual assignment of microbiota in captive tiger (*Panthera tigris*) and wildebeest (*Connochaetes taurinus*)

**DOI:** 10.1002/ece3.10066

**Published:** 2023-05-08

**Authors:** Franziska Zoelzer, Sebastian Schneider, Paul Wilhelm Dierkes

**Affiliations:** ^1^ Bioscience Education and Zoo Biology Goethe University Frankfurt Frankfurt am Main Germany

**Keywords:** 16S rRNA gene, abundance pattern, cluster analysis, microbiota

## Abstract

Fecal microbiota variability and individuality are well studied in humans and also in farm animals (related to diet‐ or disease‐specific influences), but very little is known for exotic zoo‐housed animals. This includes a wide range of species that differ greatly in microbiota composition and variation. For example, herbivorous species show a very similar and constant fecal microbiota over time, whereas carnivorous species appear to be highly variable in fecal microbial diversity and composition. Our objective was to determine whether species‐specific and individual‐specific clustering patterns were observed in the fecal microbiota of wildebeest (*Connochaetes taurinus*) and tigers (*Panthera tigris*). We collected 95 fecal samples of 11 animal individuals that were each sampled over eight consecutive days and analyzed those with Illumina MiSeq sequencing of the V3–V4 region of the 16SrRNA gene. In order to identify species or individual clusters, we applied two different agglomerative hierarchical clustering algorithms – a community detection algorithm and Ward's linkage. Our results showed that both, species‐specific and individual‐specific clustering is possible, but more reliable results were achieved when applying dynamic time warping which finds the optimal alignment between different time series. Furthermore, the bacterial families that distinguish individuals from each other in both species included daily occurring core bacteria (e.g., Acidaminococcaceae in wildebeests or Clostridiaceae in tigers) as well as individual dependent and more fluctuating bacterial families. Our results suggest that while it is necessary to consider multiple consecutive samples per individual, it is then possible to characterize individual abundance patterns in fecal microbiota in both herbivorous and carnivorous species. This would allow establishing individual microbiota profiles of animals housed in zoos, which is a basic prerequisite to quickly detect deviations and use microbiome analysis as a non‐invasive and cost‐effective tool in animal welfare.

## Graphical abstract

Our objective was to characterize species‐specific and individual‐specific abundance patterns in the fecal microbiota of herbivores and carnivores, represented by wildebeests (*Connochaetes taurinus*) and tigers (*Panthera tigris*). In order to identify individual variations, we applied two different clustering algorithms with and without prior dynamic time warping. Our results suggest that while it is necessary to consider multiple consecutive samples per individual, it is then possible to characterize individual abundance patterns in fecal microbiota in both herbivorous and carnivorous species.

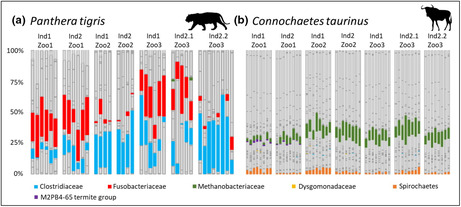

## 1 INTRODUCTION

In recent years, much research has been conducted to analyze the composition and diversity of gastrointestinal microorganisms and their impact and interaction with the host organism for various animal species as well as for phylogenetic and dietary groups (Koh et al., 2016; Ley et al., 2008; Milani et al., 2020; Nelson et al., 2015; Sanna et al., 2019; Youngblut et al., 2019). In addition to simply characterizing the species‐specific microbiome, other questions include whether microbiomes remain stable or are subject to fluctuations over time, the frequency at which these fluctuations occur, and which bacterial taxa are affected by them. Particularly in the early years of microbiome research, some studies proposed an individual long‐term stable microbiome in humans, with some bacterial taxa being persistent over a yearlong sampling interval (Björk et al., 2019; Faith et al., 2013; Hildebrand et al., 2021; Martínez et al., 2013; Schloissnig et al., 2013). In contrast, other longitudinal studies suggest that the individual human microbiome is highly variable over time (Caporaso et al., 2011; Olsson et al., 2022). Especially intra‐individual variation seems to outweigh inter‐individual variation with regard to daily fluctuation, as the majority of bacterial taxa show great shifts in abundance. Furthermore, high‐abundant taxa seem to express less variation than low‐abundant taxa and the extent of variation is constant over time (Vandeputte et al., 2021; Zoelzer et al., 2021). In contrast, there are few time‐series data on the natural variation in the fecal microbiome in various animal species. The studies conducted here (primarily on farm animals) mostly refer to the influence of dietary changes (Butowski et al., 2019; Lyu et al., 2018), impact and courses of diseases (Ayoub et al., 2022; Mamun et al., 2020) or the development of juvenile animals (Amin & Seifert, 2021; Guevarra et al., 2019; Wang et al., 2019). Only some studies refer to natural oscillations of different bacterial taxa (Björk et al., 2022; Rojas et al., 2023), finding evidence for diurnal rhythmicity in microbial diversity and composition, e.g. in dairy cows (Shaani et al., 2018) or meerkats (Risely et al., 2021).

For this reason, our study examined the daily course of the fecal microbiota in two animal species with fundamentally different digestive systems, namely tiger (*Panthera tigris*) and wildebeest (*Connochaetes taurinus*) in order to identify natural microbial abundance patterns. These two species are especially suited for time series analyses for several reasons. First, ruminants depend on bacterial fermentation to digest cellulose and, therefore, show a high microbial fecal diversity whereas carnivores with a less complex digestive system have a lower microbial diversity (Guo et al., 2020; Milani et al., 2020; Vital et al., 2015). Second, ruminants seem to have a high similarity in their microbiota, whereas the microbiota of felids is highly variable (Petri et al., 2013; Snelling et al., 2019; Zoelzer et al., 2021). Here, we survey the fecal microbiotas of tigers and wildebeest and determine whether they are species‐and individual‐specific, and whether specific bacterial families can help distinguish the different groupings.

Cluster algorithms are now widely used not only in social (Hoffman et al., 2018) or technological (Faloutsos et al., 1999) but also in biological and health‐related (Bhar et al., 2022; Fell & Wagner, 2000) network analysis. The fecal microbiota can also be considered a network in which the microbial composition of a sample represents the nodes and the distance between the samples and the respective edges of the network. In this network, closely connected nodes form a community that shares only a few edges with neighboring communities. To identify microbial communities, we used two different agglomerative hierarchical clustering algorithms—a community detection algorithm and Ward's linkage. Community detection tries to find groups of nodes that are highly connected to each other forming a cluster while Ward's linkage is based on the distance between clusters, aiming to minimize the variance within a cluster (Newman, 2004; Ward, 1963). In order to enhance the clustering results, we utilized dynamic time warping to synchronize the time series datasets and adjust for any discrepancies in sampling points. As a few studies have used dynamic time warping followed by clustering algorithms to measure the similarity between individual time series or to identify the abundance pattern of bacterial taxa over time (Armoni & Borenstein, 2022; Muinck & Trosvik, 2018; Ponziani et al., 2022), we would like to extend this approach. After clustering the individual time series, we try to identify the correct species or individual based on the microbiota composition.

Applying this approach to individual time series data of two species, we developed two hypotheses. First, we expect a clear species‐specific clustering due to the previously described significant differences in fecal microbiota composition and diversity between carnivore and herbivore species. Second, individual‐specific microbiota clustering works more reliably in tigers than in wildebeests because the herbivore microbiota is too stable within individuals to identify characteristic individual variation. Nevertheless, if not only species‐specific but also individual abundance patterns can be detected in different bacterial families and thus an individual can be identified over a time variable microbiota, this leads to several future application areas. In zoo animal husbandry, animal welfare plays a major role, e.g. in veterinary care. Using an individual fecal microbiota profile, deviations from natural fluctuations can be detected easily, inexpensively, and non‐invasively. This would provide an additional and easily accessible monitoring tool for the health of zoo animals.

## 2 MATERIALS AND METHODS

### 2.1 Sample collection

In the period from May 2018 to November 2020, 95 fecal samples were collected from 11 individuals, six tiger, and five wildebeests, housed in five German zoos (Table S1). The collection plan included a time series of eight consecutive days in which one fecal sample per day and individual was collected if available (Table A1). At least two individuals per species were sampled from each zoo. Due to sample availability, we included a total of two time series from three individuals (one tiger and two wildebeests) in the analysis to capture possible temporal variation in the microbiota. Sampling was performed non‐invasively by animal caretakers during the daily enclosure cleaning routine. The samples were immediately transferred to sterile cryotubes and stored in liquid nitrogen until further processing. We followed the EAZA research standard guidelines for the care and use of animals.

Further preparation of the samples was carried out by StarSEQ GmbH in Mainz, Germany. First, the samples were homogenized (Precellys® Evolution Homogenizer, Bertin Instruments, Rockville, USA) and subsequently DNA extraction was performed using the QIAamp® PowerFecal DNA Kit (Qiagen, Hilden, Germany). A NanoDrop spectrophotometer (Thermofisher, Massachusetts, USA) was used to measure the DNA concentration.

### 2.2 16S rRNA gene sequencing and data processing

At StarSEQ GmbH, the V3–V4 region of the 16S rRNA gene was sequenced by a dual‐index strategy based on the protocol of (Caporaso et al., 2012) with minor modifications. Amplicons were generated by a single‐step of 33 cycles using the primer combination 341f and 806bR (Apprill et al., 2015; Takahashi et al., 2014). The final library was sequenced on the Illumina MiSeq platform in paired‐end mode (300 nt) with a 25% PhiX control library. Samples were analyzed according to the QIIME 2 pipeline (Bolyen et al., 2019). As described in previous work (Zoelzer et al., 2021), DADA2 (Callahan et al., 2016) was applied to determine amplicon sequence variants (ASVs), and a phylogenetic tree was constructed for all sequences using MAFFT sequence alignments (Katoh et al., 2002). Low abundance features that are covered by less than 10 sequences, chloroplast, and mitochondrial sequences were removed from the dataset. Taxonomic assignment of ASVs was performed using a pre‐trained Naive Bayes classifier (Bokulich et al., 2018) based on the SILVA 138 full‐length database (Quast et al., 2013). The following statistical analyses were performed in R version 3.4.1 (R Core Team, 2020) using the packages vegan (Oksanen et al., 2019) and FSA (Ogle et al., 2022). To test for individual and interspecific differences in the microbial composition, ANOSIM test was performed on dissimilarity matrices with Bray‐Curtis distances. Differences in microbial richness between and within species were tested using ANOVA, followed by a post hoc pairwise t‐Test with Bonferroni correction.

**FIGURE 1:**
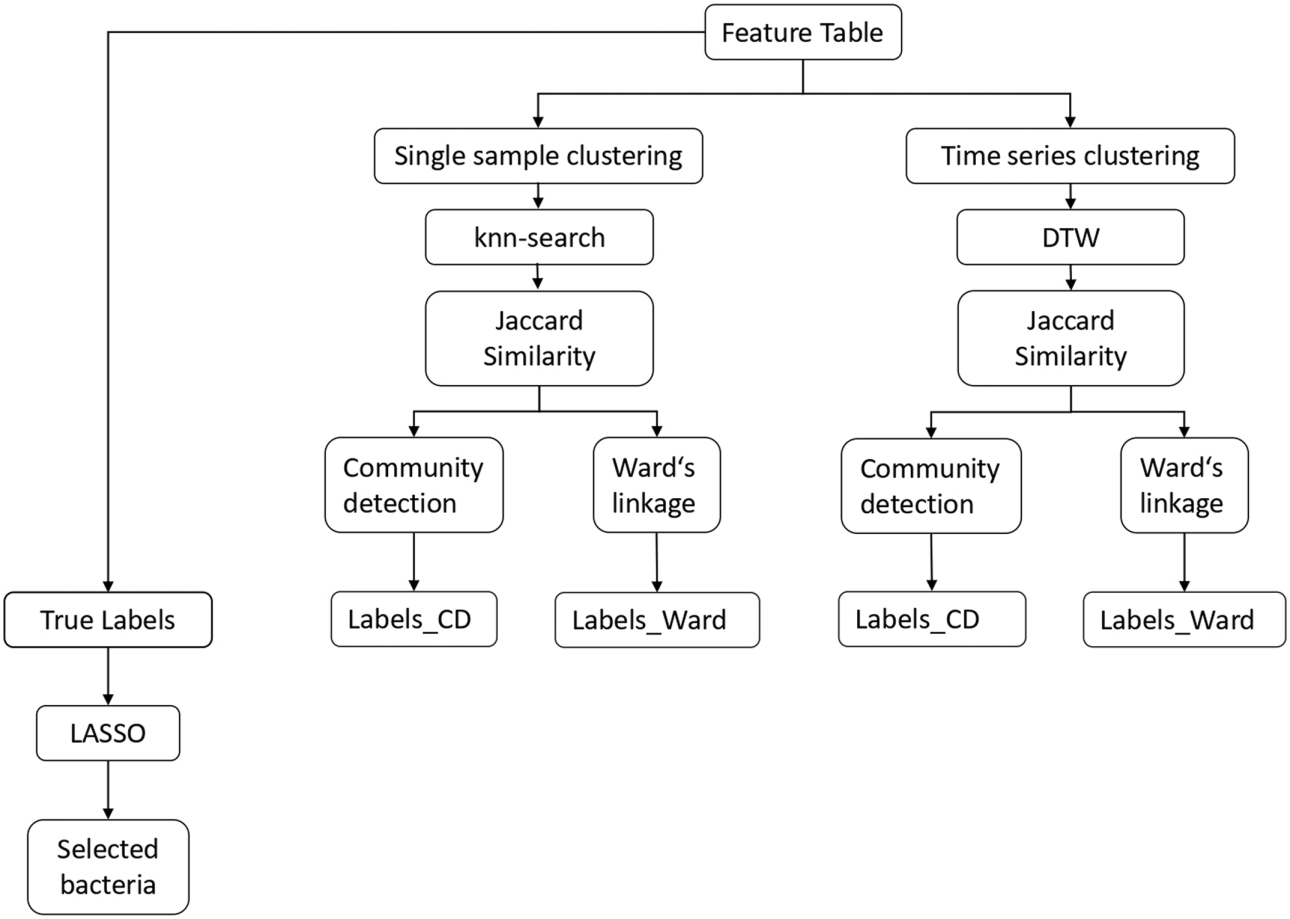
Workflow of the clustering pipeline including single and time series clustering. Starting from a feature table, both methods rely on a similarity matrix as input for the two clustering algorithms, Ward's linkage and community detection. As output, the samples within a cluster are characterized by specific labels. In addition, the true labels (known species or individuals) were used as input to the LASSO algorithm to identify individual‐ and species‐specific bacterial families.

**FIGURE 2:**
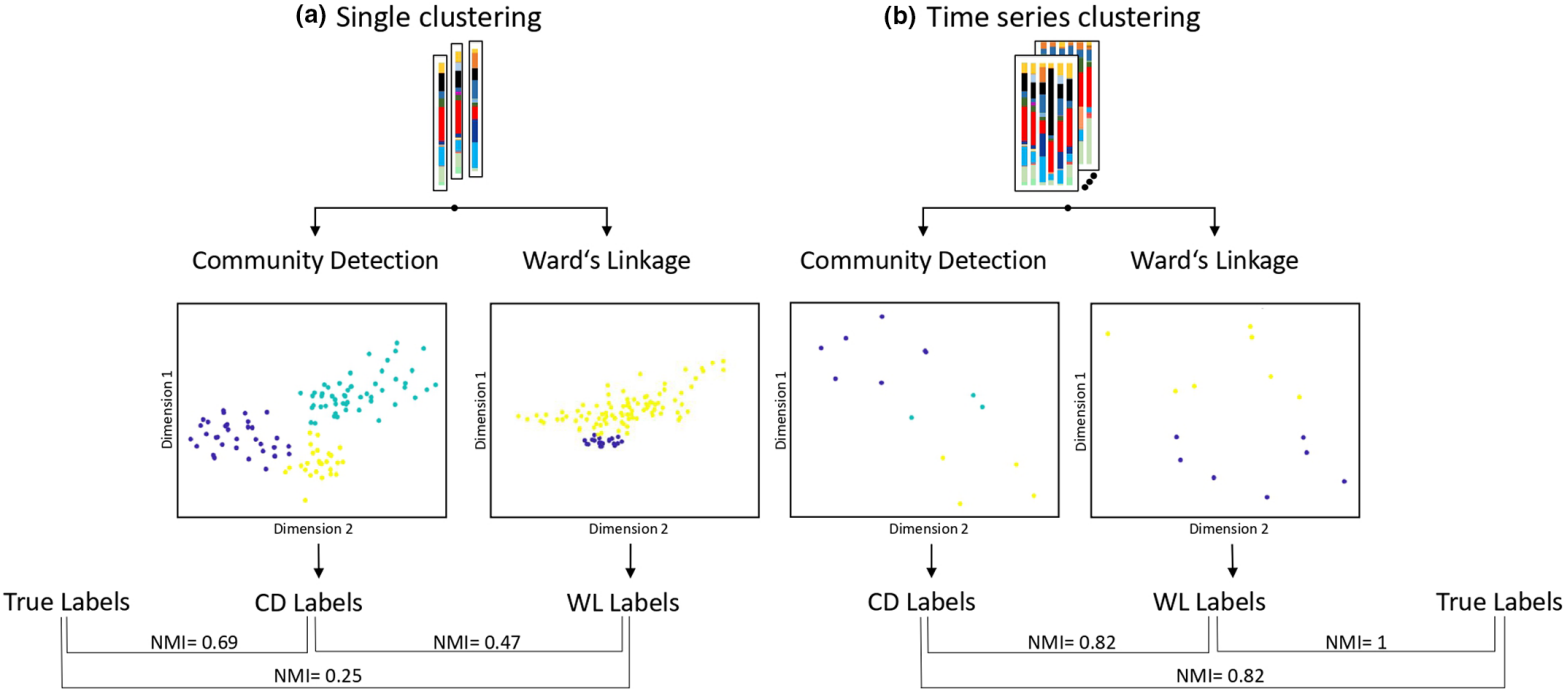
Comparison of the two clustering approaches. (a) For the single clustering approach, each sample is considered individually. The microbial composition of each sample as a single data set is used here as input for clustering. (b) For the time series clustering, a whole time series of each individual consisting of up to eight samples are used as input data for the clustering pipeline. In both approaches, the output cluster labels are compared with each other as well as with the true labels (correct species or individual per sample) by calculating the NMI value.

Most methods for time series analysis require that the time intervals between samples are equidistant, which can be difficult if the animals (mainly carnivores) do not reliably defecate on a daily basis. In this study, we solved the problem using a combined approach of dynamic time warping followed by a clustering algorithm to ensure the comparability of individual time series data and contrast the results with a method using k‐nearest‐neighbor as the classifier (Figure 1). The whole clustering pipeline is implemented in Matlab version 9.11 (The MathWorks Inc., 2020) using the software CASE (Schneider et al., 2022). Accordingly, we applied two different approaches to cluster the data (Figure 2). On the one hand, all samples were clustered individually (Single clustering). This was done by using k‐nearest‐neighbor‐search (Friedman et al., 1977) which determines for each object Ni (fecal sample) its k‐nearest neighbors with the smallest Euclidean distance and creates a distance matrix which serves as an input for the subsequent creation of a Jaccard similarity matrix. On the other hand, samples of the same individual were combined as a time series (Time series clustering) and first compared using dynamic time warping (DTW). The DTW algorithm (Paliwal et al., 1982; Sakoe & Chiba, 1978) is designed to compare two time series by calculating the Euclidean distance between them. To achieve this, each element of the two time series (or columns for matrices) is repeated until the Euclidean distance is minimized. The output is a distance matrix, which again is used to create a Jaccard similarity matrix.

In both cases, Ward's linkage and a community detection algorithm are applied to the dataset. Ward's linkage is a type of hierarchical cluster analysis technique that involves evaluating the distance between two clusters through the linkage function. This function is computed by measuring the increase in error sum of squares (ESS) that occurs when two clusters are merged into one. Ward's method aims to minimize the increase in ESS during each clustering step by selecting the most appropriate clustering steps (Ward, 1963). Here, the number of clusters was determined automatically by estimating the most consistent cluster solution in the dendrogram. Cluster solutions with two or less clusters were ignored except for the species‐specific clustering, as two clusters were to be expected. Furthermore, we used a community detection algorithm (Newman, 2004) as implemented in the software CASE (Schneider et al., 2022). This agglomerative hierarchical clustering algorithm groups vertices into clusters. It results in a hierarchical dendrogram, where at the beginning each vertex is considered as a separate community. As the algorithm progresses, it merges pairs of communities together based on the number of edges connecting their vertices, resulting in clusters with many internal edges and relatively few edges connecting vertices from different clusters (Fortunato, 2010).

To check the reliability of the cluster results, the normalized mutual information (NMI) was calculated. The NMI compares the determined labels of Ward's linkage and community detection with each other and outputs a value between 0 and 1, where 1 corresponds to an optimal match. Additionally, to evaluate the clustering in terms of individual discrimination, the NMI was also calculated against the true label. In this case, the true label refers to either the correct species (wildebeest or tiger) of a sample or the correct individual within a species. This label is used within the pipeline to compare the clustering results with the true and correct results. The true labels could be clearly determined because the associated individual was known when the fecal samples were collected. The features (bacterial families) that best describe the differences between the species and individuals in the dataset were calculated from the true labels using the LASSO algorithm (Tibshirani, 1996). LASSO is a type of shrinkage method that automatically reduces the influence of less relevant features by making them smaller and less significant. Additionally, it can perform variable selection by setting irrelevant variables to zero. The whole pipeline was performed on the entire dataset as well as on the wildebeest‐ and tiger‐specific datasets to test for intraspecific variation.

## 3 RESULTS

In total, we analyzed 95 fecal samples of five wildebeests and six tigers, performing Illumina MiSeq paired‐end sequencing of the V3–V4 region of the 16S rRNA gene. After preprocessing, the dataset consisted of 5,662,914 sequences (5836–230,928 sequences per sample) with an average of 59,610 sequences per sample. We found a significantly higher species richness in wildebeests (ANOVA statistic: *F* = 137.10, *p* < .001) than in tigers and very different microbial composition between these two species (ANOSIM statistic: *R* = .89, *p* < .001, number of permutations: 999, distance = “bray”). Within the respective species, the individuals analyzed had a significantly different microbiota, with some overlap (Wildebeest ANOSIM statistic: *R* = .18, *p* = .001; Tigers ANOSIM statistic: *R* = .29, *p* = .003, number of permutations: 999, distance = “bray”).

### 3.1 Species‐specific clustering of the microbiota

**FIGURE A1:**
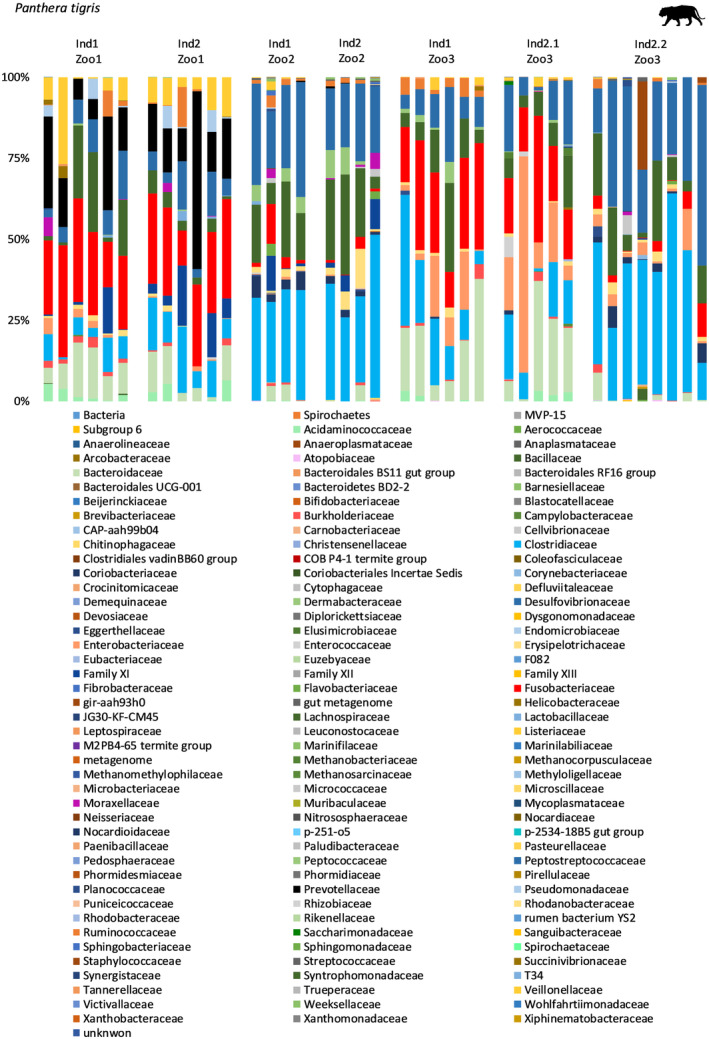
Visualization of the cluster solutions for both approaches via t‐SNE in two dimensions.

We found the highest support and strongest clustering of samples by species using dynamic time warping prior to clustering with Ward's linkage. However, when using just single samples without dynamic time warping, species‐specific clustering was not observed and samples were given incorrect species assignments (Figure A1). In the best scenario, a species‐specific assignment of all individual samples would result in two cluster solutions—wildebeests and tigers. This was not possible with either Ward's linkage (NMI_Ward/True_ = 0.25) or community detection algorithm (NMI_Com/True_ = 0.69) (Table 1). When applying dynamic time warping to the dataset which synchronizes the time series samples for individuals, the overall quality of clustering increased. Regarding species discrimination, both algorithms reached comparable results (NMI_Ward/Com_ = 0.82). While community detection (NMI_Com/True_ = 0.82) resulted in three clusters, in which all wildebeest individuals fall into one cluster and the tigers were split up into two clusters, Ward's linkage led to a correct assignment of species (NMI_Ward/True_ = 1) (Figure A1).

**TABLE 1:** Clustering results of single and time series clustering.

	NMI (com/Ward)	NMI (Ward/True_Ind_)	NMI (com/True_Ind_)	Number cluster (Ward_Ind_)	Number cluster (Com_Ind_)	NMI (com/Ward)	NMI (Ward/True_Spe_)	NMI (com/True_Spe_)	Number cluster (Ward_Spe_)	Number cluster (Com_Spe_)
**Single clustering**										
Total						0.47	0.25	0.69	2	3
Tiger	0.68	0.18	0.01	4	2					
Wilde‐beest	0.81	0.39	0.54	3	4					
**Time series clustering**										
Total						0.82	1	0.82	2	3
Tiger	0.64	0.51	0.95	3	7					
Wilde‐beest	1	0.82	0.82	6	6					

*Note*: The table shows the reliability of both algorithms (NMI) to each other and against the true species/individual label. Furthermore, the number of calculated clusters for species detection (Spe) as well as for individual discrimination (Ind) is represented. The upper part of the table represents the results for the single clustering approach and the lower part shows the results for the time series clustering. For individual discrimination, a minimum of three cluster solutions is required while for species detection results with two cluster solutions are allowed.

### 3.2 Individual‐specific clustering of the microbiota

We then sought out to test whether our clustering approaches could detect individual‐specific clustering of the microbiota in tigers and wildebeest. Due to significant differences in microbial diversity and composition between the two species, the whole dataset was separated into species‐specific sets to analyze individual differences within both species. For individual differentiation within the species, the optimal result would consist of six cluster solutions for the tigers and five for the wildebeests, one per individual. Comparing the labels determined by single clustering with the true individual labels, a low level of consistency was found (Table 1). For wildebeest, we found that the best results were achieved using community detection (NMI_Com/True_ = 0.54) which resulted in four different clusters (Figure A1). In contrast, a correct individual assignment within the tigers (NMI_Ward/True_ = 0.18, NMI_Com/True_ = 0.01) was not possible using the single clustering approach. When dynamic time warping was applied prior to clustering, individual discrimination was improved. For wildebeests, both algorithms (NMI_Ward/Com_ = 1) led to the same results. Except for two individuals, each time series was assigned to a separate cluster here. For tigers, individual‐specific clustering led to more reliable results, especially when applying a community detection algorithm (NMI_Ind_ = 0.95). In this case, seven clusters were calculated including one individual time series each. Different time series of the same individual, no matter if wildebeest or tiger were classified in different clusters by both algorithms in both clustering algorithms.

### 3.3 Identification of bacterial features that explain species differences

**FIGURE 3:**
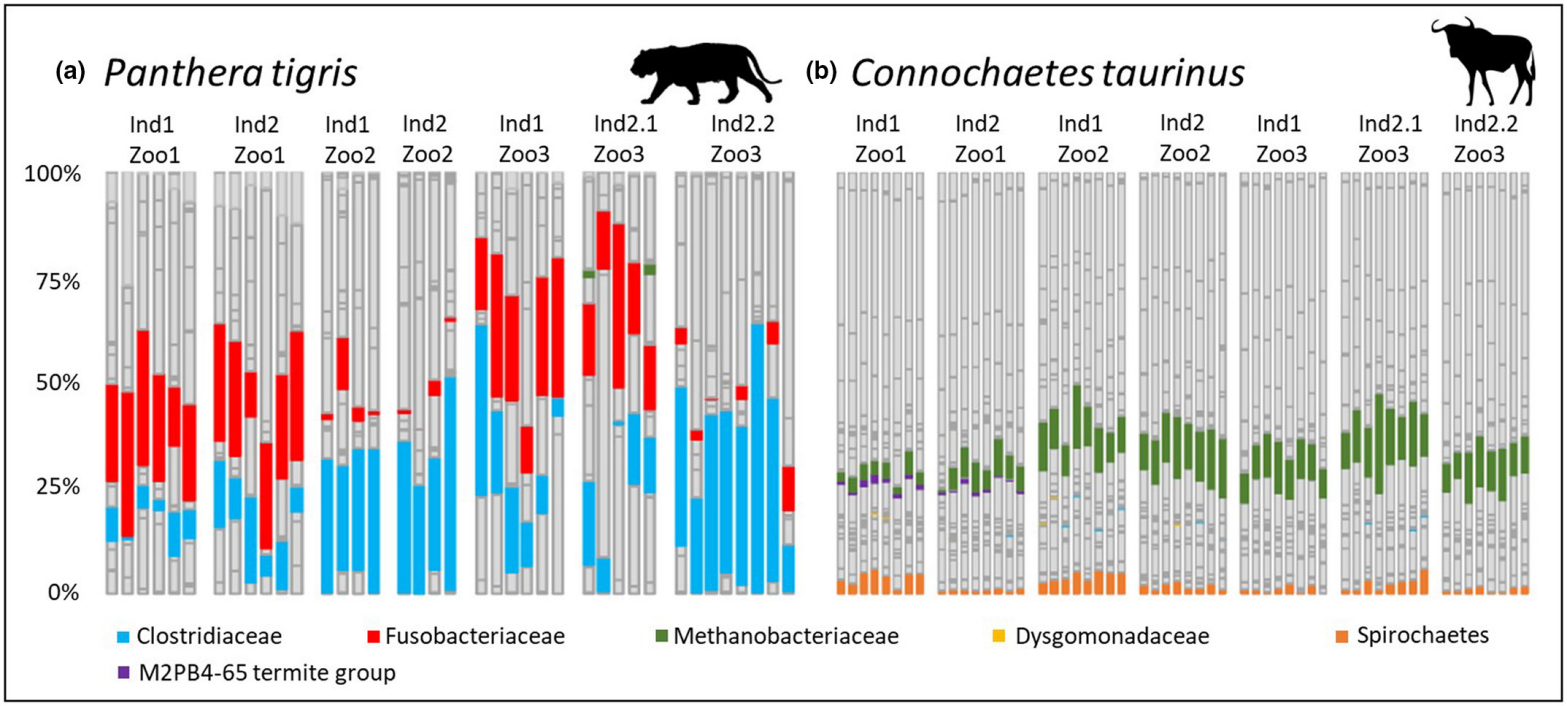
Comparison of the species‐specific bacterial families that were identified with the LASSO algorithm. (a) Tiger‐specific bacterial families as proportion of the total microbiota. (b) Wildebeest‐specific bacterial families as proportion of the total microbiota.

Using the LASSO algorithm, bacterial families leading to the specific cluster solution were identified. First, we compared all wildebeests and tigers to identify the taxa that led to a species‐specific cluster solution using the known true labels for each species. Compared to the wildebeests, the tiger‐specific bacterial families were Clostridiaceae and Fusobacteriaceae (Figure 3a). Both families are core bacteria in all individuals, meaning that they appear in each consecutive sample (Caporaso et al., 2011). On average, Clostridiaceae constituted between 5.75 ± 3.23% and 38.09 ± 14.95% to the microbiota of the seven tiger datasets. Within the microbiota of three individuals (Ind1_Zoo2, Ind2_Zoo2, Ind2.2_Zoo3) this family had a share of more than 24% on average. Fusobacteriaceae constituted between 2.12 ± 1.89% and 25.26 ± 6.64% on average to the tigers' microbiota. This family either occurred in larger proportions of more than 20% of the average microbiota or is represented by very small proportions (<5%). Considering daily time intervals, both species‐specific bacterial families seemed to be subject to larger fluctuations as can be seen in Ind2.1_Zoo3. Here, the average proportion of Clostridiaceae increased from 11.81 ± 6.68% to 38.09 ± 14.95% and that of Fusobacteriaceae decreased from 20.46 ± 9.38% to 3.21 ± 3.24% within 2 days.

In contrast to these results, wildebeest‐specific bacteria accounted for a much lower average proportion of the individual microbiota (Figure 3b). The major wildebeest‐specific bacteria were Methanobacteriaceae and the phylum Spirochaetes. Methanobacteriaceae as a core bacterial family in all individuals occurred on average between 3.28 ± 1.13% and 13.19 ± 4.28% in each dataset. Spirochaetes constituted from 0.95 ± 0.20% to 4.24 ± 1.07% to the wildebeest microbiota and were also a core bacterial phylum in all individuals except for one. Dysgomonadaceae and Clostridiaceae both occurred in <0.2% of all datasets. Nevertheless, Clostridiaceae were a persistent family within three individuals, being present in at least two consecutive sampling days. Dysgomonadaceae, as a core bacterial family in Ind1_Zoo2, constituted on average only 0.15 ± 0.05% to the respective microbiota. Finally, the M2PB4–65 termite group only occurred in minor proportions in both individuals of Zoo1 (Ind1 = 1.13 ± 0.45%, Ind2 = 0.69 ± 0.34%) but act as a core taxon in both of them.

### 3.4 Identification of bacterial features that explain individual differences

**FIGURE 4:**
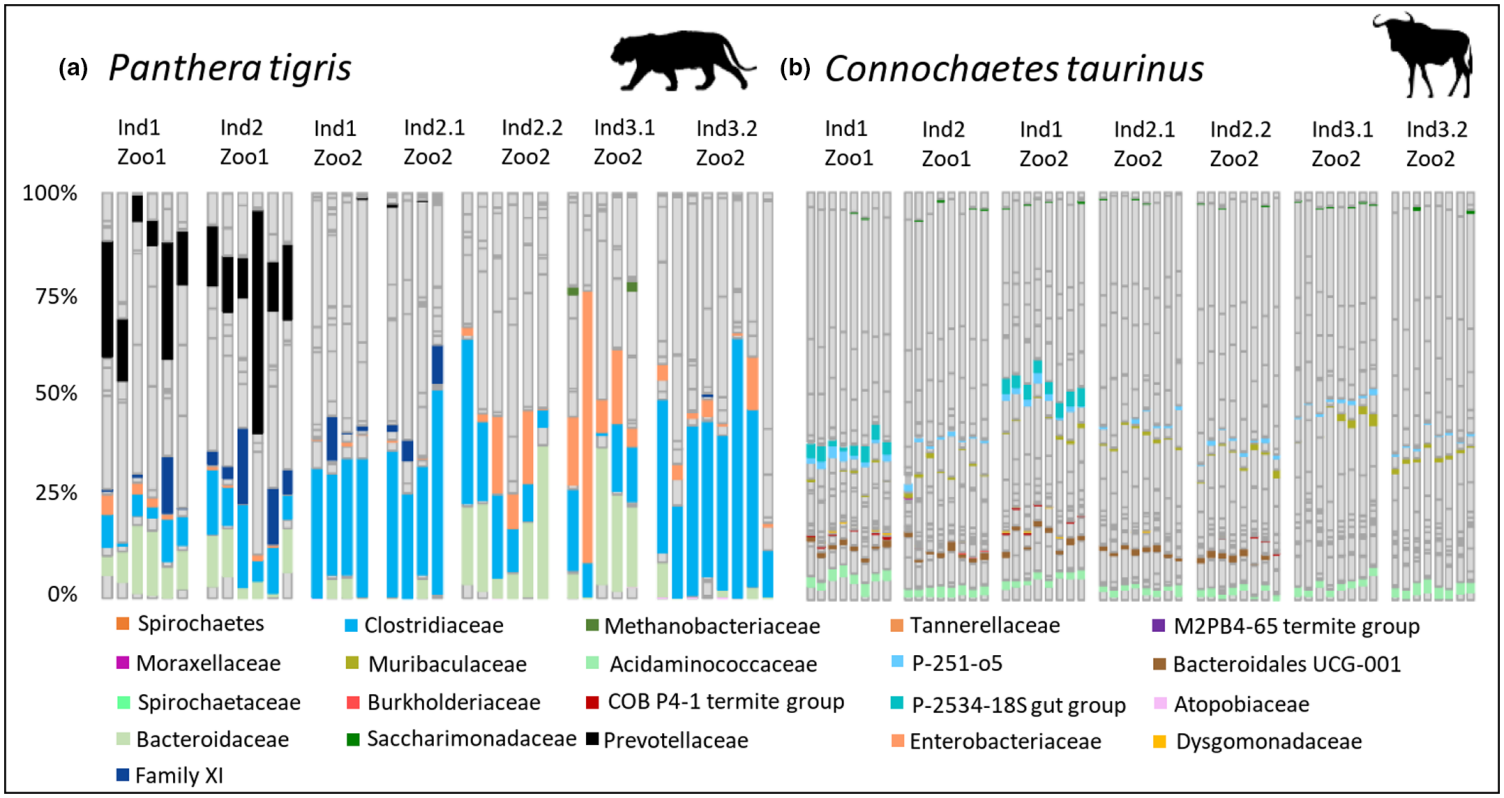
Comparison of the individual‐specific bacterial families that were identified with the LASSO algorithm. (a) Individual‐specific bacterial families identified within all tiger individuals as proportion of the total microbiota. (b) Individual‐specific bacterial families identified within all wildebeest individuals as proportion of the total microbiota.

**FIGURE A2:**
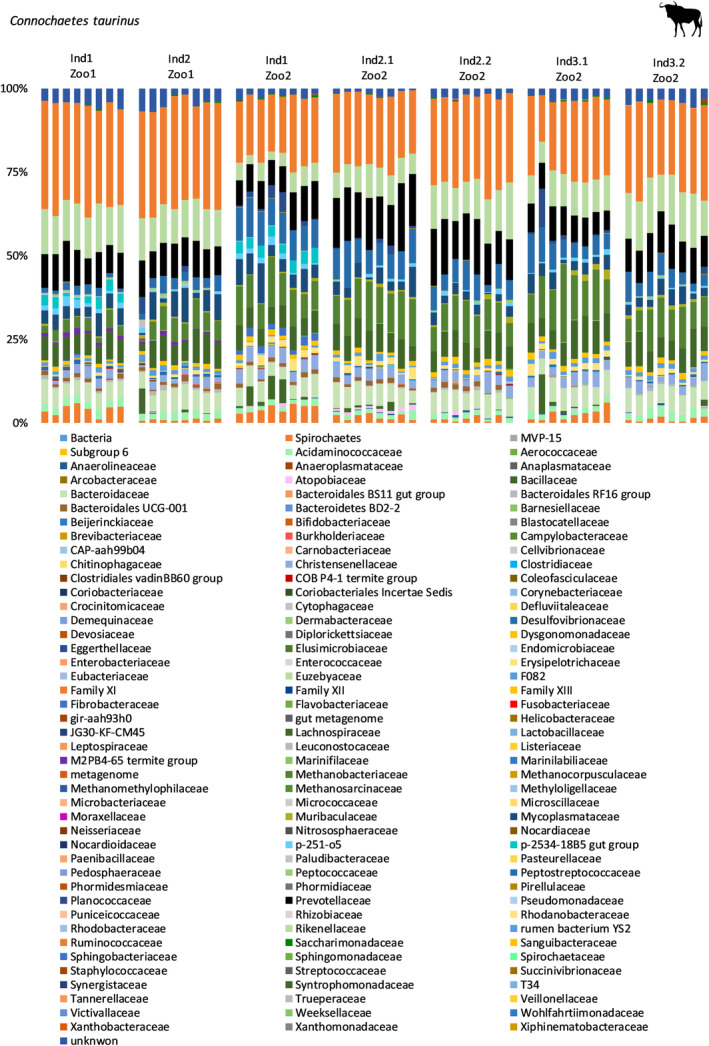
Fecal microbiota composition of all sampled tiger and wildebeest individuals.

Second, the same approach was used to identify bacteria that are responsible for the clustering of tiger individuals. The feature selection revealed seven bacterial families: Atopobiaceae, Bacteroidaceae, Clostridiaceae, Enterobacteriaceae, Family XI, Methanobacteriaceae, and Prevotellaceae. Even though Bacteroidaceae, Clostridiaceae, and Enterobacteriaceae occurred daily in almost all individuals, individuals could be distinguished based on specific bacterial combinations and fluctuations. The microbiota composition of individuals from the same zoo is often very similar (Figure 4a). For example, Prevotellaceae only appeared in greater proportions as a core taxon in both individuals from Zoo1 (Ind1_Zoo1: 16.46 ± 9.25%, Ind2_Zoo1: 20.70 ± 15.49%) and otherwise only occurred in smaller proportions within individuals from Zoo2 (Ind1_Zoo2: 0.35 ± 0.48%, Ind2_Zoo1: 0.93 ± 1.45%). Additionally, the individuals from Zoo1 showed a low average proportion of Enterobacteriaceae being persistent members (Ind1_Zoo1: 1.75 ± 1.71%, Ind2_Zoo1: 0.52 ± 0.48%) while this family was a core member in all other individuals. Both individuals from Zoo2 also showed a lower proportion of Enterobacteriaceae (Ind1_Zoo2: 1.28 ± 1.82%, Ind2_Zoo2: 1.17 ± 1.95%), but can be distinguished from the former individuals because of a 10 times lower average amount of Bacteroidaceae. The greatest proportion of Enterobacteriaceae was found in Ind1_Zoo3 (8.28 ± 7.70%) and Ind2.1_Zoo3 (22.84 ± 22.58%) whereas this family is less abundant in Ind2.2_Zoo3 (3.50 ± 3.83%). Even if the examined tiger individuals from the same habitat seemed to be similar (Figure A2), the time series clustering via the community detection algorithm showed that individuals can be identified via individual abundance patterns in their microbiota composition.

For individual discrimination within the wildebeests, the LASSO algorithm identified 12 bacterial taxa: Bacteroidales UCG‐001, Muribaculaceae, p‐251‐o5, p‐2534–18S gut group, Acidaminococcaceae, Saccharimonadaceae, Burkholderiaceae, Moraxellaceae, Spirochaetaceae, COB P4‐1 termite group, Dysgomonadaceae and Tannerellaceae (Figure 4b). Even if most of these were core bacteria in many individuals, their proportion on the total microbiota is much lower compared to the tiger‐specific bacteria. For example, Acidaminococcaceae on average only occurred between 1.46 ± 0.40% in Ind2.1_Zoo2 and 2.60 ± 0.49% in Ind3.2_Zoo2. Two individuals could not be discriminated from each other and were classified in the same cluster (Ind1_Zoo1, Ind1_Zoo2). These two individuals were the only ones in which p‐2534–18S was found in the fecal microbiota. Because of the wildebeest microbial composition being very similar across all animals (Figure A2), individual clustering was based on low‐frequent but steadily occurring bacterial families. Nevertheless, it is possible to identify individual microbiota within the wildebeests using a dynamic time‐warping approach followed by a community detection clustering algorithm.

## 4 DISCUSSION

### 4.1 Evaluation of clustering algorithms

The aim of this study was to characterize abundance patterns in species‐specific as well as in individual‐specific fecal microbiota in an herbivore and a carnivore species over eight consecutive sampling days. Therefore, we chose two hierarchical agglomerative clustering algorithms that are able to perform reliably on smaller and biological datasets (Girvan & Newman, 2002; Terada, 2013; Yang et al., 2016). The ability of Ward's linkage to correctly assign species was limited when applying the single clustering pipeline. Possible limitations of this cluster algorithm include unequal distribution of sample sizes, the occurrence of outlier samples, and an elliptical rather than circular distribution of samples (Everitt et al., 2010). The former does not apply to the dataset used, but the latter two points could be a reason for the inadequate results of this algorithm since outlier samples, in particular, are well possible due to daily fluctuations in the microbiota. The limitations described above may also result in the inability to unambiguously classify species using the community detection algorithm in single clustering, resulting in three cluster solutions instead of two.

In order to improve clustering results, we synchronized the time series samples per individual with dynamic time warping to balance uneven sampling points. This approach has recently become the focus of meaningful interpretation of longitudinal data sets. By aligning different time series, data sets become comparable and temporal effects on the microbiota between or within individuals can be evaluated (Armoni & Borenstein, 2022; Lugo‐Martinez et al., 2019; Muinck & Trosvik, 2018). The alignment of the individual time series to each other also led to a significant improvement in the cluster solutions in our study. Regarding the time series clustering, Ward's linkage resulted in a correct species‐specific clustering when comparing the labels to the true species labels. The community detection algorithm also resulted in a correct assignment of wildebeests but failed to correctly identify all tiger samples. Nevertheless, this result confirms our assumption and previous results due to the greater variability in the microbiota within this species (Figure A2) (Karmacharya et al., 2019; Ning et al., 2020; Zoelzer et al., 2021).

### 4.2 Individual‐specific clustering based on longitudinal data

Both species differed significantly in alpha diversity as well as in microbial composition, which was to be expected and has already been shown in several studies (Nishida & Ochman, 2018; Zhu et al., 2018). To avoid biasing the cluster algorithms with these fundamental differences, we split the dataset into two species‐specific datasets to test for individual variation in the microbiota. Similar to species‐specific clustering, the high variability in the microbiota of tigers was probably responsible for the fact that no individuals can be assigned via single clustering. Both algorithms formed non‐specific clusters, represented through a very low NMI when each sample is considered individually. Nevertheless, we managed to considerably improve the results of individual clustering by applying dynamic time warping to the individual samples and thus synchronizing the individual time series. The combination of up to eight samples per time series provided the cluster algorithm with more information to process and thus led to more reliable clustering. In addition, outlier samples which negatively influence the algorithms (Everitt et al., 2010), could be correctly classified into the natural microbial oscillations of an individual, which was not possible when considering each sample individually.

Limitations to successful individual assignment arose from comparing different time periods of the same individual. While eight consecutive samples were sufficient to characterize the individual microbiota for exactly this period, more samples are needed to close the gap between longer sampling intervals. A reason for that might be the temporal dynamics of the fecal microbiota. Even if we found individual abundance patterns in the microbiota, the actual rhythm could be longer than 8 days and would not be fully captured in this study. Furthermore, other influencing factors that are known to shape the microbiota as diet, habitat, or seasonal shifts can lead to ongoing changes in the bacterial composition. In zoos, wildebeests are typically sustained on a diet of hay, alfalfa, or grass, which remains fairly consistent throughout the year. In contrast, the diet of tigers is more variable and can undergo daily changes in meat origin or preparation, such as whole‐body or sheared meat. Studies have shown that felids' fecal microbiota is significantly affected by changes in diet and its composition, particularly alterations in the ratio of carbohydrates to protein (Bermingham et al., 2017; Butowski et al., 2019; Wernimont et al., 2020). To control for the habitat as an influencing factor, we also compared the clustering results with the true zoo‐related labels. However, we did not obtain meaningful results and therefore excluded the habitat factor from further analysis. Another reason for the distinct time series clustering of the same individual might be seasonal adjustments of the microbiota. Seasonal shifts in microbiota composition have been reported previously in bisons (*Bison bison*) (Bergmann et al., 2015), musk deer (*Moschus* spp) (Jiang et al., 2021), primates (Baniel et al., 2021; Sawada et al., 2022), and also giant pandas (*Ailuropoda melanoleuca*) (Xue et al., 2015). Given all these variables, it is clear that even in a controlled habitat such as a zoo, changes or variations in feeding schedules or enclosure design must be well documented and taken into account when comparing longitudinal data sets.

### 4.3 LASSO identified features for cluster

Once we assigned an individual microbiota to tigers and wildebeests, the next step was to identify the bacteria being responsible for this distinction. Species‐specific clustering was mainly influenced by Fusobacteriaceae, Clostridiaceae, Methanobacteriaceae, and Spirochaetes with the first two being tiger‐specific and the last mentioned being wildebeest‐specific bacteria. Both Fusobacteriaceae and Clostridiaceae occurred within each individual and in each sample. This is in line with the results of our previous comparative study in which we were able to show that exactly these two families are the major families in many carnivore species (Zoelzer et al., 2021). Both taxa are involved in protein metabolism and the production of short‐chain fatty acids (Basson et al., 2016; Bermingham et al., 2017; Vital et al., 2014) and are more abundant in species with a high‐fat diet such as different predators (Bragg et al., 2020; Milani et al., 2020; Vital et al., 2015). In contrast, Methanobacteriaceae and Spirochaetes were core bacteria in nearly all wildebeest individuals. Herbivores depend on microbial fermentation for carbohydrate digestion and, for example, Spirochaetes are capable of producing short‐chain fatty acids from polysaccharide intake (Angelakis et al., 2019; de Filippo et al., 2010). Accordingly, this phylum has been found less in carnivores but more often in many herbivore species (Thingholm et al., 2021; Zoelzer et al., 2021). Methanobacteriaceae play an important role especially in ruminants as wildebeests, as they use the end products of microbial fermentation, CO_2_ or H_2_, as substrates to produce methane. This avoids an excessive increase in H_2_ partial pressure in the rumen and the ambient factors for microbial digestive enzymes can be kept constant (Balch et al., 1979; Delzenne & Cani, 2011; Hook et al., 2010; Morgavi et al., 2010; Patra et al., 2017). All things considered, taxa that are responsible for species‐specific clustering are mostly core bacteria that are involved in the specific herbivore or carnivore digestion process.

Additionally, we identified bacterial families that are necessary to characterize the individual microbiota of tigers and wildebeests. On the one hand, tiger individuals could be distinguished by core bacterial families such as Clostridiaceae, Enterobacteriaceae, and Bacteroidaceae. These are either involved in protein digestion or are members of the normal carnivore fecal microbiota (Kerr et al., 2013; Panasevich et al., 2015; Schwab & Gänzle, 2011; Xue et al., 2015). Even though these occurred daily, they showed host‐specific fluctuations and can thus be used to determine individual differences. On the other hand, not only variations in the core bacteria determined the individual tiger microbiota but also individual‐specific bacterial families. These accounted for either a large (e.g. Prevotellaceae) or small proportion (e.g., Atopobiaceae) of the total microbiota and are known to undergo fluctuations in the carnivore fecal microbiota (Guo et al., 2020; Ley et al., 2008).

In contrast to tigers, wildebeests showed a very uniform microbiota but nevertheless individual clustering and thus an assignment to an individual microbiota based on specific bacteria was possible. Accordingly, variation of high abundant bacterial families could not be responsible for individual differences as in tigers, but the distinctions were in low abundant bacterial families. Nevertheless, the pattern remained the same. General rumen‐specific bacteria such as Acidaminococcaceae or the phylum Spirochaetes (Savin et al., 2022; Snelling et al., 2019) and bacterial families contributing to milk production in cattle such as Muribaculaceae and p‐251‐o5 (Boggio et al., 2021; Kodithuwakku et al., 2022) were considered to distinguish individual wildebeests. In contrast, bacterial families that only occurred in single individuals or that varied greatly among individuals were found by the LASSO algorithm to cluster individual microbiota (e.g. Burkholderiaceae, Moraxellaceae, p‐2534–18B5 gut group). Overall, it can be concluded that there are individual abundance patterns of bacterial families, both in animal species with a highly variable as well as in species with a very constant microbiota. These are a combination of core bacteria of the respective species and other individual or zoo‐specific families in varying abundance.

## 5 CONCLUSION

To the best of our knowledge, this is the first study focusing on the characterization of individual oscillations in the microbiota of two species, applying different clustering algorithms to the sequencing data. With this research, we were able to show that two species with completely different diets exhibit both species‐specific and individual abundance patterns in the fecal microbiota over the period of 1 week. Thus, we confirm our first hypothesis that a species‐specific microbiota can be detected by the applied clustering pipeline. In addition, we showed that these results were considerably improved if time series data are considered and evaluated via dynamic time warping and community detection algorithm. Our second hypothesis that individual abundance patterns are more reliably detected in tigers than in wildebeest due to the more variable microbiota, cannot be clearly confirmed. Although the individual identification of the tigers resulted in a slightly higher NMI value when compared to the true labels, individual differences can also be detected within the very constant fecal microbiota of the wildebeests, with only marginally inferior cluster solutions compared to the tigers. Bacterial families that are responsible for individual clustering follow a similar pattern in both species. Individual abundance patterns are subject to a combination of species‐specific core and individual‐specific highly‐fluctuating bacterial families.

From a methodological point of view, it can be implied for further studies that the use and interpretation of single individual samples or collective group samples is critical. Our results show that the microbiota of wildebeest and tiger are also subject to fluctuations that can only be captured through time series data. As one of the main tasks of zoos is to continuously improve animal welfare, they can benefit from an individualized microbial profile of some animal species that show e.g. special dietary requirements or increased stress susceptibility. The non‐invasive sampling is easy to integrate into the daily routine and the evaluation is cost‐effective. Accordingly, a fecal microbial profile is an easy‐to‐use method to continuously monitor individuals and, if necessary, to perform individual treatments or adjustments in the feeding schedule.

## AUTHOR CONTRIBUTIONS

**Franziska Zoelzer:** Conceptualization (lead); formal analysis (lead); methodology (lead); writing – original draft (lead). **Sebastian Schneider:** Conceptualization (supporting); software (lead); writing – review and editing (equal). **Paul W. Dierkes:** Funding acquisition (lead); supervision (lead); writing – review and editing (equal).

## CONFLICT OF INTEREST STATEMENT

The authors declare that they have no competing interests.

## Supporting information


Table S1

## Data Availability

DNA sequences: NCBI SRA BioProject PRJNA912880 (all samples) and PRJNA716130 (BioSamples: SAMN18396313, SAMN18396314, SAMN18396324–SAMN18396328, SAMN18396336, SAMN18396346, SAMN18396347, SAMN18396350, SAMN18396351, SAMN18396498, SAMN18396525, SAMN18396526, SAMN18396532, SAMN18396533, SAMN18396536–SAMN18396539, SAMN18396542, SAMN18396543, SAMN18396652–SAMN18396654, SAMN18396657–SAMN18396659, SAMN18396666–SAMN18396668, SAMN18396681–SAMN18396683, SAMN18396686–SAMN18396688, SAMN18396691–SAMN18396693, SAMN18396696–SAMN18396698, SAMN18396701–SAMN18396703).

## APPENDIX A

**TABLE A1:** Metadata of all samples analyzed in this study including the species, sex, age and sampling date.

Individual_ID	Species	Sampling_Day	Sex	Year	Month	Age
Ind1_Zoo1	*Connochaetes_taurinus*	1	Female	2020	2	2
Ind1_Zoo1	*Connochaetes_taurinus*	2	Female	2020	2	2
Ind1_Zoo1	*Connochaetes_taurinus*	3	Female	2020	2	2
Ind1_Zoo1	*Connochaetes_taurinus*	4	Female	2020	2	2
Ind1_Zoo1	*Connochaetes_taurinus*	5	Female	2020	2	2
Ind1_Zoo1	*Connochaetes_taurinus*	6	Female	2020	2	2
Ind1_Zoo1	*Connochaetes_taurinus*	7	Female	2020	2	2
Ind1_Zoo1	*Connochaetes_taurinus*	8	Female	2020	2	2
Ind1_Zoo2	*Connochaetes_taurinus*	1	Male	2020	7	18
Ind1_Zoo2	*Connochaetes_taurinus*	2	Male	2020	7	18
Ind1_Zoo2	*Connochaetes_taurinus*	3	Male	2020	7	18
Ind1_Zoo2	*Connochaetes_taurinus*	4	Male	2020	7	18
Ind1_Zoo2	*Connochaetes_taurinus*	5	Male	2020	7	18
Ind1_Zoo2	*Connochaetes_taurinus*	6	Male	2020	8	18
Ind1_Zoo2	*Connochaetes_taurinus*	7	Male	2020	8	18
Ind1_Zoo2	*Connochaetes_taurinus*	8	Male	2020	8	18
Ind2.1_Zoo2	*Connochaetes_taurinus*	1	Female	2020	7	17
Ind2.1_Zoo2	*Connochaetes_taurinus*	2	Female	2020	7	17
Ind2.1_Zoo2	*Connochaetes_taurinus*	3	Female	2020	7	17
Ind2.1_Zoo2	*Connochaetes_taurinus*	4	Female	2020	7	17
Ind2.1_Zoo2	*Connochaetes_taurinus*	5	Female	2020	7	17
Ind2.1_Zoo2	*Connochaetes_taurinus*	6	Female	2020	8	17
Ind2.1_Zoo2	*Connochaetes_taurinus*	7	Female	2020	8	17
Ind2.1_Zoo2	*Connochaetes_taurinus*	8	Female	2020	8	17
Ind2.2_Zoo2	*Connochaetes_taurinus*	1	Female	2020	12	17
Ind2.2_Zoo2	*Connochaetes_taurinus*	2	Female	2020	12	17
Ind2.2_Zoo2	*Connochaetes_taurinus*	3	Female	2020	12	17
Ind2.2_Zoo2	*Connochaetes_taurinus*	4	Female	2020	12	17
Ind2.2_Zoo2	*Connochaetes_taurinus*	5	Female	2020	12	17
Ind2.2_Zoo2	*Connochaetes_taurinus*	6	Female	2020	12	17
Ind2.2_Zoo2	*Connochaetes_taurinus*	7	Female	2020	12	17
Ind2.2_Zoo2	*Connochaetes_taurinus*	8	Female	2020	12	17
Ind2_Zoo1	*Connochaetes_taurinus*	1	Male	2020	2	3
Ind2_Zoo1	*Connochaetes_taurinus*	2	Male	2020	2	3
Ind2_Zoo1	*Connochaetes_taurinus*	3	Male	2020	2	3
Ind2_Zoo1	*Connochaetes_taurinus*	4	Male	2020	2	3
Ind2_Zoo1	*Connochaetes_taurinus*	5	Male	2020	2	3
Ind2_Zoo1	*Connochaetes_taurinus*	6	Male	2020	2	3
Ind2_Zoo1	*Connochaetes_taurinus*	7	Male	2020	2	3
Ind2_Zoo1	*Connochaetes_taurinus*	8	Male	2020	2	3
Ind3.1_Zoo2	*Connochaetes_taurinus*	1	Female	2020	7	1
Ind3.1_Zoo2	*Connochaetes_taurinus*	2	Female	2020	7	1
Ind3.1_Zoo2	*Connochaetes_taurinus*	3	Female	2020	7	1
Ind3.1_Zoo2	*Connochaetes_taurinus*	4	Female	2020	7	1
Ind3.1_Zoo2	*Connochaetes_taurinus*	5	Female	2020	7	1
Ind3.1_Zoo2	*Connochaetes_taurinus*	6	Female	2020	8	1
Ind3.1_Zoo2	*Connochaetes_taurinus*	7	Female	2020	8	1
Ind3.1_Zoo2	*Connochaetes_taurinus*	8	Female	2020	8	1
Ind3.2_Zoo2	*Connochaetes_taurinus*	1	Female	2020	12	1
Ind3.2_Zoo2	*Connochaetes_taurinus*	2	Female	2020	12	1
Ind3.2_Zoo2	*Connochaetes_taurinus*	3	Female	2020	12	1
Ind3.2_Zoo2	*Connochaetes_taurinus*	4	Female	2020	12	1
Ind3.2_Zoo2	*Connochaetes_taurinus*	5	Female	2020	12	1
Ind3.2_Zoo2	*Connochaetes_taurinus*	6	Female	2020	12	1
Ind3.2_Zoo2	*Connochaetes_taurinus*	7	Female	2020	12	1
Ind3.2_Zoo2	*Connochaetes_taurinus*	8	Female	2020	12	1
Ind1_Zoo1	*Panthera_tigris*	1	Female	2018	5	10
Ind1_Zoo1	*Panthera_tigris*	2	Female	2018	5	10
Ind1_Zoo1	*Panthera_tigris*	3	Female	2018	5	10
Ind1_Zoo1	*Panthera_tigris*	5	Female	2018	5	10
Ind1_Zoo1	*Panthera_tigris*	6	Female	2018	5	10
Ind1_Zoo1	*Panthera_tigris*	8	Female	2018	5	10
Ind1_Zoo2	*Panthera_tigris*	2	Male	2020	12	8
Ind1_Zoo2	*Panthera_tigris*	3	Male	2020	12	8
Ind1_Zoo2	*Panthera_tigris*	4	Male	2020	12	8
Ind1_Zoo2	*Panthera_tigris*	5	Male	2020	12	8
Ind1_Zoo3	*Panthera_tigris*	1	Female	2018	6	17
Ind1_Zoo3	*Panthera_tigris*	2	Female	2018	6	17
Ind1_Zoo3	*Panthera_tigris*	3	Female	2018	6	17
Ind1_Zoo3	*Panthera_tigris*	4	Female	2018	6	17
Ind1_Zoo3	*Panthera_tigris*	5	Female	2018	6	17
Ind1_Zoo3	*Panthera_tigris*	8	Female	2018	6	17
Ind2.1_Zoo3	*Panthera_tigris*	1	Male	2018	6	8
Ind2.1_Zoo3	*Panthera_tigris*	2	Male	2018	6	8
Ind2.1_Zoo3	*Panthera_tigris*	3	Male	2018	6	8
Ind2.1_Zoo3	*Panthera_tigris*	4	Male	2018	6	8
Ind2.1_Zoo3	*Panthera_tigris*	5	Male	2018	6	8
Ind2.2_Zoo3	*Panthera_tigris*	1	Male	2020	11	10
Ind2.2_Zoo3	*Panthera_tigris*	2	Male	2020	11	10
Ind2.2_Zoo3	*Panthera_tigris*	3	Male	2020	11	10
Ind2.2_Zoo3	*Panthera_tigris*	4	Male	2020	11	10
Ind2.2_Zoo3	*Panthera_tigris*	5	Male	2020	11	10
Ind2.2_Zoo3	*Panthera_tigris*	6	Male	2020	11	10
Ind2.2_Zoo3	*Panthera_tigris*	7	Male	2020	11	10
Ind2.2_Zoo3	*Panthera_tigris*	8	Male	2020	11	10
Ind2_Zoo1	*Panthera_tigris*	1	Male	2018	5	7
Ind2_Zoo1	*Panthera_tigris*	2	Male	2018	5	7
Ind2_Zoo1	*Panthera_tigris*	3	Male	2018	5	7
Ind2_Zoo1	*Panthera_tigris*	5	Male	2018	5	7
Ind2_Zoo1	*Panthera_tigris*	7	Male	2018	5	7
Ind2_Zoo1	*Panthera_tigris*	8	Male	2018	5	7
Ind2_Zoo2	*Panthera_tigris*	1	Female	2020	12	7
Ind2_Zoo2	*Panthera_tigris*	3	Female	2020	12	7
Ind2_Zoo2	*Panthera_tigris*	5	Female	2020	12	7
Ind2_Zoo2	*Panthera_tigris*	6	Female	2020	12	7
