# Supplementary material for: Time series cluster analysis reveals individual assignment of microbiota in captive tiger (Panthera tigris) and wildebeest (Connochaetes taurinus)
Source: Ecol Evol. 2023 May 8;13(5):e10066. doi: 10.1002/ece3.10066 (PMC10166651; doi:10.1002/ece3.10066)
Supplement: Supplementary file 1 — Table S1 [file ECE3-13-e10066-s001.docx]

Appendix:

Table S1: Metadata of all analyzed samples. For each sample the respective species, sex, and sampling period is shown.

| **Individual_ID** | **Species** | **Sampling_Day** | **Sex** | **Year** | **Month** | **Age** |
| --- | --- | --- | --- | --- | --- | --- |
| Ind1_Zoo1 | *Connochaetes_taurinus* | 1 | Female | 2020 | 2 | 2 |
| Ind1_Zoo1 | *Connochaetes_taurinus* | 2 | Female | 2020 | 2 | 2 |
| Ind1_Zoo1 | *Connochaetes_taurinus* | 3 | Female | 2020 | 2 | 2 |
| Ind1_Zoo1 | *Connochaetes_taurinus* | 4 | Female | 2020 | 2 | 2 |
| Ind1_Zoo1 | *Connochaetes_taurinus* | 5 | Female | 2020 | 2 | 2 |
| Ind1_Zoo1 | *Connochaetes_taurinus* | 6 | Female | 2020 | 2 | 2 |
| Ind1_Zoo1 | *Connochaetes_taurinus* | 7 | Female | 2020 | 2 | 2 |
| Ind1_Zoo1 | *Connochaetes_taurinus* | 8 | Female | 2020 | 2 | 2 |
| Ind1_Zoo2 | *Connochaetes_taurinus* | 1 | Male | 2020 | 7 | 18 |
| Ind1_Zoo2 | *Connochaetes_taurinus* | 2 | Male | 2020 | 7 | 18 |
| Ind1_Zoo2 | *Connochaetes_taurinus* | 3 | Male | 2020 | 7 | 18 |
| Ind1_Zoo2 | *Connochaetes_taurinus* | 4 | Male | 2020 | 7 | 18 |
| Ind1_Zoo2 | *Connochaetes_taurinus* | 5 | Male | 2020 | 7 | 18 |
| Ind1_Zoo2 | *Connochaetes_taurinus* | 6 | Male | 2020 | 8 | 18 |
| Ind1_Zoo2 | *Connochaetes_taurinus* | 7 | Male | 2020 | 8 | 18 |
| Ind1_Zoo2 | *Connochaetes_taurinus* | 8 | Male | 2020 | 8 | 18 |
| Ind2.1_Zoo2 | *Connochaetes_taurinus* | 1 | Female | 2020 | 7 | 17 |
| Ind2.1_Zoo2 | *Connochaetes_taurinus* | 2 | Female | 2020 | 7 | 17 |
| Ind2.1_Zoo2 | *Connochaetes_taurinus* | 3 | Female | 2020 | 7 | 17 |
| Ind2.1_Zoo2 | *Connochaetes_taurinus* | 4 | Female | 2020 | 7 | 17 |
| Ind2.1_Zoo2 | *Connochaetes_taurinus* | 5 | Female | 2020 | 7 | 17 |
| Ind2.1_Zoo2 | *Connochaetes_taurinus* | 6 | Female | 2020 | 8 | 17 |
| Ind2.1_Zoo2 | *Connochaetes_taurinus* | 7 | Female | 2020 | 8 | 17 |
| Ind2.1_Zoo2 | *Connochaetes_taurinus* | 8 | Female | 2020 | 8 | 17 |
| Ind2.2_Zoo2 | *Connochaetes_taurinus* | 1 | Female | 2020 | 12 | 17 |
| Ind2.2_Zoo2 | *Connochaetes_taurinus* | 2 | Female | 2020 | 12 | 17 |
| Ind2.2_Zoo2 | *Connochaetes_taurinus* | 3 | Female | 2020 | 12 | 17 |
| Ind2.2_Zoo2 | *Connochaetes_taurinus* | 4 | Female | 2020 | 12 | 17 |
| Ind2.2_Zoo2 | *Connochaetes_taurinus* | 5 | Female | 2020 | 12 | 17 |
| Ind2.2_Zoo2 | *Connochaetes_taurinus* | 6 | Female | 2020 | 12 | 17 |
| Ind2.2_Zoo2 | *Connochaetes_taurinus* | 7 | Female | 2020 | 12 | 17 |
| Ind2.2_Zoo2 | *Connochaetes_taurinus* | 8 | Female | 2020 | 12 | 17 |
| Ind2_Zoo1 | *Connochaetes_taurinus* | 1 | Male | 2020 | 2 | 3 |
| Ind2_Zoo1 | *Connochaetes_taurinus* | 2 | Male | 2020 | 2 | 3 |
| Ind2_Zoo1 | *Connochaetes_taurinus* | 3 | Male | 2020 | 2 | 3 |
| Ind2_Zoo1 | *Connochaetes_taurinus* | 4 | Male | 2020 | 2 | 3 |
| Ind2_Zoo1 | *Connochaetes_taurinus* | 5 | Male | 2020 | 2 | 3 |
| Ind2_Zoo1 | *Connochaetes_taurinus* | 6 | Male | 2020 | 2 | 3 |
| Ind2_Zoo1 | *Connochaetes_taurinus* | 7 | Male | 2020 | 2 | 3 |
| Ind2_Zoo1 | *Connochaetes_taurinus* | 8 | Male | 2020 | 2 | 3 |
| Ind3.1_Zoo2 | *Connochaetes_taurinus* | 1 | Female | 2020 | 7 | 1 |
| Ind3.1_Zoo2 | *Connochaetes_taurinus* | 2 | Female | 2020 | 7 | 1 |
| Ind3.1_Zoo2 | *Connochaetes_taurinus* | 3 | Female | 2020 | 7 | 1 |
| Ind3.1_Zoo2 | *Connochaetes_taurinus* | 4 | Female | 2020 | 7 | 1 |
| Ind3.1_Zoo2 | *Connochaetes_taurinus* | 5 | Female | 2020 | 7 | 1 |
| Ind3.1_Zoo2 | *Connochaetes_taurinus* | 6 | Female | 2020 | 8 | 1 |
| Ind3.1_Zoo2 | *Connochaetes_taurinus* | 7 | Female | 2020 | 8 | 1 |
| Ind3.1_Zoo2 | *Connochaetes_taurinus* | 8 | Female | 2020 | 8 | 1 |
| Ind3.2_Zoo2 | *Connochaetes_taurinus* | 1 | Female | 2020 | 12 | 1 |
| Ind3.2_Zoo2 | *Connochaetes_taurinus* | 2 | Female | 2020 | 12 | 1 |
| Ind3.2_Zoo2 | *Connochaetes_taurinus* | 3 | Female | 2020 | 12 | 1 |
| Ind3.2_Zoo2 | *Connochaetes_taurinus* | 4 | Female | 2020 | 12 | 1 |
| Ind3.2_Zoo2 | *Connochaetes_taurinus* | 5 | Female | 2020 | 12 | 1 |
| Ind3.2_Zoo2 | *Connochaetes_taurinus* | 6 | Female | 2020 | 12 | 1 |
| Ind3.2_Zoo2 | *Connochaetes_taurinus* | 7 | Female | 2020 | 12 | 1 |
| Ind3.2_Zoo2 | *Connochaetes_taurinus* | 8 | Female | 2020 | 12 | 1 |
| Ind1_Zoo1 | *Panthera_tigris* | 1 | Female | 2018 | 5 | 10 |
| Ind1_Zoo1 | *Panthera_tigris* | 2 | Female | 2018 | 5 | 10 |
| Ind1_Zoo1 | *Panthera_tigris* | 3 | Female | 2018 | 5 | 10 |
| Ind1_Zoo1 | *Panthera_tigris* | 5 | Female | 2018 | 5 | 10 |
| Ind1_Zoo1 | *Panthera_tigris* | 6 | Female | 2018 | 5 | 10 |
| Ind1_Zoo1 | *Panthera_tigris* | 8 | Female | 2018 | 5 | 10 |
| Ind1_Zoo2 | *Panthera_tigris* | 2 | Male | 2020 | 12 | 8 |
| Ind1_Zoo2 | *Panthera_tigris* | 3 | Male | 2020 | 12 | 8 |
| Ind1_Zoo2 | *Panthera_tigris* | 4 | Male | 2020 | 12 | 8 |
| Ind1_Zoo2 | *Panthera_tigris* | 5 | Male | 2020 | 12 | 8 |
| Ind1_Zoo3 | *Panthera_tigris* | 1 | Female | 2018 | 6 | 17 |
| Ind1_Zoo3 | *Panthera_tigris* | 2 | Female | 2018 | 6 | 17 |
| Ind1_Zoo3 | *Panthera_tigris* | 3 | Female | 2018 | 6 | 17 |
| Ind1_Zoo3 | *Panthera_tigris* | 4 | Female | 2018 | 6 | 17 |
| Ind1_Zoo3 | *Panthera_tigris* | 5 | Female | 2018 | 6 | 17 |
| Ind1_Zoo3 | *Panthera_tigris* | 8 | Female | 2018 | 6 | 17 |
| Ind2.1_Zoo3 | *Panthera_tigris* | 1 | Male | 2018 | 6 | 8 |
| Ind2.1_Zoo3 | *Panthera_tigris* | 2 | Male | 2018 | 6 | 8 |
| Ind2.1_Zoo3 | *Panthera_tigris* | 3 | Male | 2018 | 6 | 8 |
| Ind2.1_Zoo3 | *Panthera_tigris* | 4 | Male | 2018 | 6 | 8 |
| Ind2.1_Zoo3 | *Panthera_tigris* | 5 | Male | 2018 | 6 | 8 |
| Ind2.2_Zoo3 | *Panthera_tigris* | 1 | Male | 2020 | 11 | 10 |
| Ind2.2_Zoo3 | *Panthera_tigris* | 2 | Male | 2020 | 11 | 10 |
| Ind2.2_Zoo3 | *Panthera_tigris* | 3 | Male | 2020 | 11 | 10 |
| Ind2.2_Zoo3 | *Panthera_tigris* | 4 | Male | 2020 | 11 | 10 |
| Ind2.2_Zoo3 | *Panthera_tigris* | 5 | Male | 2020 | 11 | 10 |
| Ind2.2_Zoo3 | *Panthera_tigris* | 6 | Male | 2020 | 11 | 10 |
| Ind2.2_Zoo3 | *Panthera_tigris* | 7 | Male | 2020 | 11 | 10 |
| Ind2.2_Zoo3 | *Panthera_tigris* | 8 | Male | 2020 | 11 | 10 |
| Ind2_Zoo1 | *Panthera_tigris* | 1 | Male | 2018 | 5 | 7 |
| Ind2_Zoo1 | *Panthera_tigris* | 2 | Male | 2018 | 5 | 7 |
| Ind2_Zoo1 | *Panthera_tigris* | 3 | Male | 2018 | 5 | 7 |
| Ind2_Zoo1 | *Panthera_tigris* | 5 | Male | 2018 | 5 | 7 |
| Ind2_Zoo1 | *Panthera_tigris* | 7 | Male | 2018 | 5 | 7 |
| Ind2_Zoo1 | *Panthera_tigris* | 8 | Male | 2018 | 5 | 7 |
| Ind2_Zoo2 | *Panthera_tigris* | 1 | Female | 2020 | 12 | 7 |
| Ind2_Zoo2 | *Panthera_tigris* | 3 | Female | 2020 | 12 | 7 |
| Ind2_Zoo2 | *Panthera_tigris* | 5 | Female | 2020 | 12 | 7 |
| Ind2_Zoo2 | *Panthera_tigris* | 6 | Female | 2020 | 12 | 7 |
